# Recent progress of nanotechnology-based theranostic systems in cancer treatments

**DOI:** 10.20892/j.issn.2095-3941.2020.0510

**Published:** 2021-06-15

**Authors:** Ying Xue, Yuting Gao, Fanling Meng, Liang Luo

**Affiliations:** 1 National Engineering Research Center for Nanomedicine, College of Life Science and Technology, Huazhong University of Science and Technology, Wuhan 430074, China; 2 Hubei Key Laboratory of Bioinorganic Chemistry and Materia Medica, School of Chemistry and Chemical Engineering, Huazhong University of Science and Technology, Wuhan 430074, China; 3 Research Institute of Huazhong University of Science and Technology in Shenzhen, Shenzhen 518057, China

**Keywords:** Theranostics, cancer therapy, diagnosis, nanoparticles, nanotechnology

## Abstract

Theranostics that integrates therapy and diagnosis in one system to achieve accurate cancer diagnosis and treatment has attracted tremendous interest, and has been recognized as a potential breakthrough in overcoming the challenges of conventional oncotherapy. Nanoparticles are ideal candidates as carriers for theranostic agents, which is attributed to their extraordinary physicochemical properties, including nanoscale sizes, functional properties, prolonged blood circulation, active or passive tumor targeting, specific cellular uptake, and in some cases, excellent optical properties that ideally meet the needs of phototherapy and imaging at the same time. Overall, with the development of nanotechnology, theranostics has become a reality, and is now in the transition stage of “bench to bedside.” In this review, we summarize recent progress on nanotechnology-based theranostics, i.e., nanotheranostics, that has greatly assisted traditional therapies, and has provided therapeutic strategies emerging in recent decades, as well as “cocktail” theranostics mixing various treatment modalities.

## Introduction

Cancer is still threatening the life and health of many humans. According to the latest global surveillance that includes individual records of 37.5 million patients diagnosed with cancer during 2000–2014^1^, cancer survival rates are generally rising, including some cancers with high malignancies, but the survival rates of some cancers are still far from satisfactory. For example, the 5-year net survivals of lung, liver, and pancreatic cancers in China are all below 20%. In addition, large differences in the 5-year net survivals among regions and races have been reported. Current first-line cancer therapies in the clinic, including chemotherapy, radiotherapy, and surgery, suffer from a variety of challenges, such as low tumor specificity and a high level of systemic toxicity, limited penetration through the highly dense extracellular matrix, multi-drug resistance, and inadequate clearance^2–4^. In particular, the risk of treatment failure or tumor recurrence and metastasis still increases if timely diagnosis is absent. Effective and personalized treatment strategies that integrate both cancer diagnosis and therapeutic methods are therefore needed to provide satisfactory clinical outcomes.

Strategies combining diagnosis and therapy, namely theranostics, that allow simultaneous detection of targets^3^, monitoring of drug distribution^5^, and the evaluation of therapeutic responses^6^ to achieve personalized medicine^7^ have attracted great interest. Recent rapid developments in nanotechnologies have encouraged researchers to develop nanoparticle-based transport platforms for co-delivery of diagnosis and therapeutic drugs. The small size of nanomaterials endows them with large surface areas and high drug-loading capacities, making it possible to co-deliver multiple types of therapeutic drugs and imaging agents. In addition, it has been universally acknowledged that abnormal vessels in tumor tissues induce aberrant molecular and fluid transport dynamics, so nanoparticles are able to passively accumulate in tumors following their well-known enhanced permeabilities and retention effects^8^. The introduction of targeting ligands on nanoparticles, including folate, hyaluronic acid, transferrin, aptamers, antibodies, and peptides^9^ have further improved their targeting efficiency by specific recognition between ligands and the receptors on the surface of tumor tissues. In addition to superior delivery, many nanomaterials can serve as imaging agents themselves without additional loading of imaging agents, which has been attributed to their unique physicochemical properties. For example, iron oxide can be used in magnetic resonance imaging (MRI)^10^, which means that diagnosis and treatment can be performed simultaneously rather than before or after therapy^11^. The surface plasmon resonance (SPR) effect of gold nanoparticles may also assist the overall theranostic outcomes.

Collectively, nanotechnology-assisted cancer theranostics, or cancer nanotheranostics, have many unique advantages, including passive or active accumulation in tumor tissues based on different mechanisms, and the excellent optical properties of some nanocarriers can directly participate in specific oncotherapy and diagnosis^12^, real-time imaging guided therapy, and the combination of various therapeutic and diagnostic nanoparticles can be used in a single system for synergistic therapeutic effects^5,13,14^.

**Figure 1:**
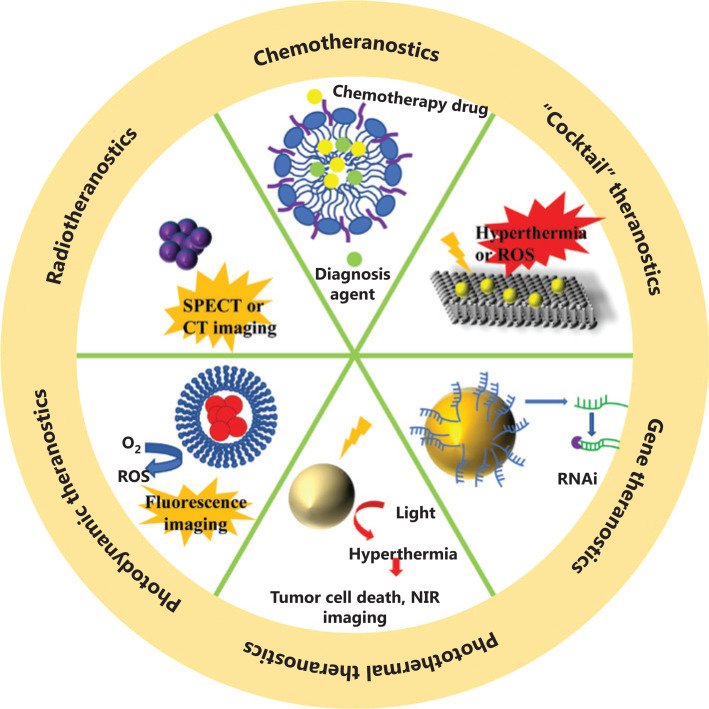
Nanotechnology-based theranostic systems for different cancer therapy methods.

In this review, we focus on the recent progress of nanotechnology-based theranostic systems that have been widely used to assist in many clinical and preclinical cancer treatments, from classic first-line therapies (chemotherapy and radiotherapy), to therapeutic strategies proposed in recent decades, such as photodynamic therapy (PDT), photothermal therapy (PTT), and gene therapy (**Figure 1**). **Table 1** summarizes some representative nanomaterials, which have been used as carriers to assist in the delivery of drugs, or as functional agents for treatments and diagnoses. Particular emphasis has been directed to the design of novel nanotheranostics systems that have the potential for simultaneous cancer diagnosis and treatment in clinical translations.

**Table 1:** Representative nanocarriers that are commonly used for nanotheranostics

Materials	Characteristics	Application	References
Liposomes	Amphiphilic	Nanocarriers for co-delivery of hydrophobic and hydrophilic drugs	^15,16^
Polymers	Biocompatible and easy to modify	Drug intelligent responses, functional coating	^3,15,17^
Black phosphorus	Large surface-area-to-volume ratio and layer-dependent bandgap	Drug carriers, photoacoustic imaging, phototherapy, photothermal therapy	^18^
Mesoporous silica	Porous structure, optical performance, easy modification and protecting drugs against multi-drug resistance	Drug carriers	^2,19^
Iron oxide	Magnetic properties and biocompatibility	MRI imaging, hyperthermia treatment	^10^
Gold	SPR effect and photothermal effect	PA imaging, Raman spectroscopy imaging, radiotherapy, phototherapy	^20–22^
Quantum dots	Non-radiative decay, superior stability	Delivery platform, photodynamic therapy, photothermal therapy	^2,23^
Graphene	Strong NIR light absorption, high surface area	Carriers, NIR fluorescence probes, PA imaging, photothermal therapy	^2,5,23^

## Nanotechnology-based chemotheranostics

Chemotherapy is one of the most widely accepted cancer treatments. However, untargeted delivery of chemotherapeutic drugs usually results in unnecessary drug accumulation in non-tumor sites, which may cause severe side effects and multi-drug resistance. Imaging-guided delivery of chemotherapeutic drugs facilitated by nanotheranostics can significantly reduce the off-target risk. In addition, use of a theranostic system can also result in timely, accurate, and noninvasive detection of early responses of chemotherapies. Overall, the application of nanotechnology-based theranostic platforms provides chemotherapy with unparalleled advantages for overcoming its long-accompanied disadvantages.

### Common nanocarriers for chemotheranostics

Organic materials, especially those from natural resources, such as liposomes^24^, hyaluronic acid (HA)^25,26^, and folic acid (FA)^27^ possess a number of superior qualities as carriers, such as good biocompatibility, low immunogenicity, and reduced administration frequency^28^. Liposomes are one of the most widely used organic drug carriers, which are spherical vesicles formed by lipid bilayers^16^. Owing to their amphiphilic nature, liposomes are able to load both hydrophobic and hydrophilic agents with high loading efficiency, while protecting them from biodegradation. Another important organic drug carrier is HA, which is a primary CD44 binding molecule. Because CD44 is overexpressed and correlated with tumor progression in many types of cancers^29^, HA has been used in nanocarriers, with preferential tumor accumulation and increased cell uptake characteristics. Moreover, the biodegradation of HA is associated with hyaluronidase and oxidative stress^17^, which are associated with the progression of tumors, so that drug release from HA is more dependent on the tumor microenvironment. FA is also widely used for targeted medicine transport. Similar to HA, FA has a high binding affinity towards folate receptors, which are highly expressed on the surface of many malignant tumors^29^. Besides HA and FA, the specific binding between antigen and antibody pairs is another important targeting strategy. Vladimir and co-workers designed antibody-directed nanoscale metal-organic frameworks^30^, which could be selectively absorbed by HER2/neu-positive cancer cells. Polymers such as polysaccharides^31^ are also commonly used as a carrier for theranostic agents.

### Nanotheranostics-assisted chemotherapy

Hou et al.^32^ developed a nanoparticle system called TCAD by conjugating D-α-tocopheryl polyethylene glycol 1000 succinate with cis-aconitic anhydride-modified doxorubicin (CAD). TCAD can self-assemble into nanoparticles with high surface areas when dissolved in aqueous solutions. Chlorine6 was loaded into TCAD to achieve fluorescence imaging-guided combined chemotherapy and photodynamic therapy (PDT). CAD was pH sensitive, and *in vitro* experiments showed that at pH 6.5 or even pH 5.5, the cumulative release of doxorubicin (DOX) and Ce6 was accelerated dramatically compared with that at pH 7.4, suggesting the enhanced release in an acidic tumor microenvironment. The combination of chemotherapy and PDT showed synergistic inhibition of tumor proliferation. Using real-time monitoring of drug tumor targeting and distribution, *in vivo* experiments showed that a group of A549 tumor-bearing mice treated with TCAD@Ce6 nanoparticles and near-infrared (NIR) irradiation showed significantly enhanced therapeutic efficiencies compared to free DOX or free Ce6.

**Figure 2:**
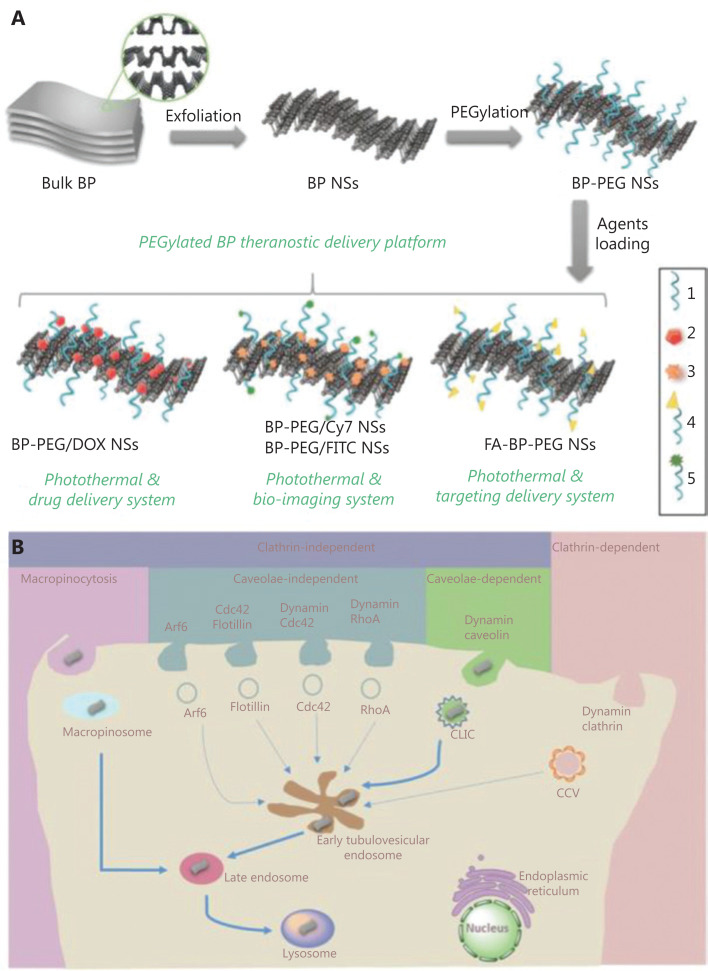
(A) Schematic representation of the PEGylated BP theranostic delivery platform (1: PEG-NH_2_, 2: DOX, 3: Cy7-NH_2_, 4: FA-PEG-NH_2_, 5: FITC-PEG-NH_2_). (B) Screening and summary of the endocytosis pathways and biological activities of PEGylated BP NSs in cancer cells. Reproduced with permission from Ref. 34.

With a specific porous structure, inorganic nanoparticles possess extremely high loading capacities compared to other materials. Horcajada et al.^33^ designed a series of porous metal-organic-framework nanocarriers with a busulfan loading capacity of up to 25 wt%, compared to 5–6 wt% of polymer nanoparticles and 0.4 wt% of liposomes. In addition, the unique optical effects of inorganic materials make them particularly suitable for synergetic theranostic applications^2^. However, unlike many organic materials, inorganic nanoparticles suffer from problems such as low biostability and biocompatibility. Tao et al.^34^ developed PEGylated black phosphorus (BP) nanosheets (NS) as a drug delivery platform for tumor theranostics (**Figure 2**). BP-based nanomaterials usually serve as efficient PDT and PTT agents. In this study, polyethylene glycol-amine (PEG-NH_2_) was used to functionalize BP NS to enhance their biocompatibility and physiological stability. The developed PEGylated BP NSs could load theranostic agents with high efficiency, such as DOX for chemotherapy and cyanine7 for *in vivo* NIR imaging. Together with the intrinsic photothermal effect of BP, these PEGylated BP NSs enable a triple-response combined theranostic strategy for cancer treatment.

## Nanotheranostics-assisted radiotherapy

With a history of over 70 years^35^, radiotherapy is another first-line cancer treatment modality, which uses high energy ionizing radiation (such as gamma rays or X-rays)^36^ to kill tumor cells. Limited by the blood-brain barrier, tumors like neuroendocrine neoplasms^37^ are less sensitive to chemotherapy, whereas radiotherapy represents an effective noninvasive treatment strategy. Radioactive iodine was among the earliest radiotherapy agents for the treatment of thyroid diseases, and has been used for other diseases such as neuroendocrine tumors and prostate cancer^35,38^. In recent years, many other types of radionuclides have attracted increased attention. Especially, high atomic number elements, including bismuth^39^, yttrium^40^, and lanthanides^41^, have been used in nanotheranostics for enhanced radiotherapy efficacies.

Iikuni^40^ and co-workers prepared ^111^In and a ^90^Y-labeled ureidosulfonamide scaffold (US) to target carbonic anhydrase-IX (CA-IX), which is a typical biomarker highly expressed in many hypoxic tumor cells. With the use of ^111^In (γ-emitter) for single photon emission computed tomography (SPECT) imaging and ^90^Y (β^-^-emitter) for radiotherapy, the labeled ureidosulfonamide scaffold could be used as targeting radiotheranostic agents. HT-29 cells, with overexpressed CA-IX during hypoxic conditions, exhibited enhanced absorption of the double-labeled radiotheranostic agents under hypoxic conditions, whereas MDA-MB-231 cells with scarce CA-IX showed significantly lower absorption of labeled US. *In vivo* experiments validated the high accumulation of ^111^In and ^90^Y-labeled US in HT-29 tumor-bearing mice, where delayed tumor growth by the radiotheranostic agent was observed, when compared with the saline group.

**Figure 3:**
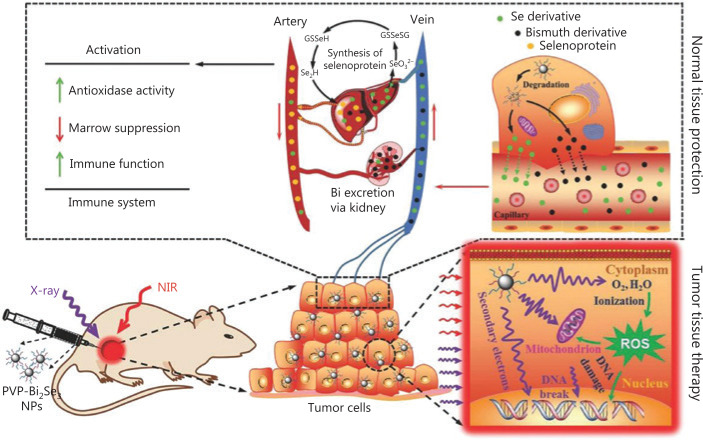
Schematic illustration of synergistic photothermal radiotherapy of tumors guided by PVP-Bi2Se3@Sec theranostic nanoparticles. Reproduced with permission by Ref. 42.

Du et al.^42^ developed new versatile Bi_2_Se_3_ nanoparticles for enhanced radiotheranostics and reduced side effects of radiation (**Figure 3**). To improve the biostability and water solubility, the nanoparticles were modified with poly(vinylpyrrolidone) (PVP) and selenocysteine (Sec). After coating with Sec and PVP, the PVP-Bi_2_Se_3_@Sec nanoparticles showed good cellular internalization ability and negligible cytotoxicity. In addition to X-ray absorption, PVP-Bi_2_Se_3_@Sec nanoparticles were also used as photothermal agents because of their high NIR absorption, enabling the combination of radiotherapy and PTT. *In vitro* experiments showed that when treated with PVP-Bi_2_Se_3_@Sec with only X-ray or 808 nm NIR irradiation, the viability values of BEL-7402 cells were 38% and 41%, respectively. In comparison, when treated with PVP-Bi_2_Se_3_@Sec with both X-ray and 808 nm NIR irradiation, the viability of BEL-7402 cells was significantly reduced to 15%, suggesting the high synergistic effect of radiotherapy and PTT. *In vivo* experiments of BEL-7402 tumor-bearing BALB/c nude mice, guided by NIR photothermal imaging, further showed that PVP-Bi2Se3@ Sec NPs plus PTT and radiotherapy had excellent therapeutic effects.

Gao et al.^43^ developed PEGylated W-doped TiO_2_ (WTO) nanoparticles, which served as radiotherapy and PTT agents, as well as contrast agents for computed tomography (CT) and photoacoustic (PA) imaging. PEGylation of nanoparticles resulted in excellent stability and dispersibility, and the optical properties of PEGylated WTO nanoparticles were not affected by extra modifications. The nanoparticles showed a concentration-dependent temperature elevation and excellent photothermal stability, with almost the same performance during several cycles of radiation. *In vitro* experiments showed the synergistic therapeutic effect involved a combination of radiotherapy and PTT, with 62% and 51% cell viabilities for PTT and radiotherapy alone, respectively, and 21% for combined treatment. An *in vivo* study, followed by both CT and PA imaging, showed significant inhibition of tumor growth by the combined therapy group, suggesting the great potential of PEGylated WTO nanoparticles as photothermal-radio theranostic agents.

## Photodynamic theranostics based on nanotechnology

With the rapid development of optical technology, nanoparticles allowing simultaneous light-induced diagnostics, imaging, as well as therapy are becoming increasingly popular in cancer theranostics^44^. As a noninvasive therapeutic strategy, PDT kills cancer cells through reactive oxygen species (ROS) generated by a photosensitizer upon irradiation, without damaging surrounding normal tissues^45,46^. In addition to the therapeutic effect, photodynamic theranostics also uses the optical characteristics of the photosensitizer or the carrier for simultaneous imaging and PDT^47^. According to the source of irradiation, photodynamic theranostics can be classified as direct irradiation photodynamic theranostics or through-energy-transfer photodynamic theranostics.

### Direct irradiation photodynamic theranostics

Targeted delivery of photosensitizers towards tumor cells is of crucial importance to the success of PDT. Nanomaterials such as quantum dots (QDs), silica, upconversion nanoparticles, and liposomes are frequently used as carriers for the co-delivery of photosensitizers and other therapeutic agents. Li et al.^48^ designed a class of semiconducting polymer nanoparticles coated with cell membranes, which could target cancer-associated fibroblasts to overcome the delivery limitation in the tumor microenvironment. Yu et al.^49^ used hollow mesoporous silica nanoparticles as a transport platform, taking advantage of their high surface areas to facilitate high drug loading.

In contrast to most reported theranostic platforms that rely on the integration of 2 or more molecular components with different functionalities in 1 delivery system, single-molecule photodynamic theranostics can avoid many problems of nanoparticles, such as limited stability, reproducibility, and restricted loading and release efficiency^50,51^. Shi et al.^52^ developed a single-molecule photodynamic theranostic agent, in which a fluorophore and a photosensitizer were conjugated by caspase-responsive peptides. The fluorescence had characteristic aggregation-induced emission (AIE)^53^, and could be released and exhibited fluorescence during tumor cell apoptosis, to indicate the death of tumor cells.

**Figure 4:**
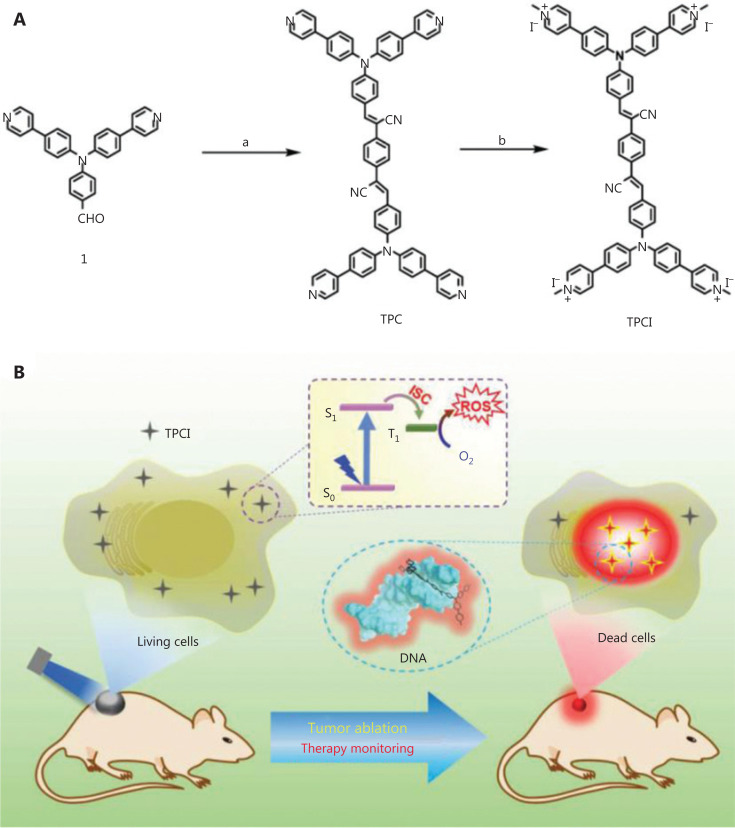
(A) Synthesis and structure of TPCI. (B) Schematic illustration of photodynamic theranostics by single molecule TPCI, which has a super-efficient photodynamic therapy effect under light irradiation, and synchronously self-illuminates cell death and tumor ablation in real time. Reproduced with permission from Ref. 54.

Compared with conjugates, Gao and co-workers^54^ developed a dual-function small molecule photosensitizer, TPCI, with intrinsic PDT efficacy and with the ability of simultaneous self-monitoring of therapeutic responses in real time (**Figure 4**). TPCI has a donor-acceptor-donor core structure with 4 terminal methylpyridinium groups, which result in TPCI with an ultrahigh singlet oxygen (^1^O_2_) quantum yield of 98.6%. In addition, TPCI has weak fluorescence in living cells before irradiation. Upon very mild irradiation, it efficiently kills cancer cells and translocates from the cytoplasm to nuclei. The binding between TPCI and chromatin activates the AIE of TPCI, enabling the real-time monitoring of cell death by TPCI itself. *In vivo* studies using several tumor-bearing mouse models validated the efficient photodynamic theranostics using single molecule TPCI.

Later, Wang et al.^55^ used liposomes to co-deliver paclitaxel (PTX) and TPCI to obtain a theranostic system with synergistic chemo-photodynamic therapy effects. The encapsulation efficiencies of liposomes were shown to be over 75%, while the loading of TPCI increased in the presence of PTX due to the interaction between the two agents. In addition, the TPCI/PTX@Lipo nanoparticle showed excellent stability with no apparent aggregation or precipitation for 2 weeks. As a theranostic agent, TPCI/PTX@Lipo is expected to provide fluorescence imaging for the direct detection of therapeutic effects. After irradiation, the fluorescence of PC3 cells treated with TPCI/PTX@Lipo and PI showed increased overlap, suggesting that TPCI/PTX@Lipo showed the therapeutic response of cell death. *In vivo* studies also showed that the synergistic anticancer effect by TPCI/PTX@Lipo was seen in treated PC3-tumor-bearing mice, which exhibited tumor ablation. Furthermore, based on the specific interaction between TPCI and DNA, the same group also developed a new molecule, TPBT, which was able to recognize double-stranded DNA (dsDNA), with a detection limit of 100 pM, as well as the ability to distinguish single nucleotide polymorphisms in a dsDNA^56^.

### Through-energy-transfer photodynamic theranostics

One of the most important limitations of PDT is insufficient tissue penetration of light. As a result, PDT is less effective for deep tissue tumors. Under these circumstances, energy transfer based on the resonance of chemiluminescence (CL) or bioluminescence (BL) has been proposed as an irradiation source to excite PDT photosensitizers. CL-involved PDT uses chemiluminescence resonance energy transfer (CRET) to stimulate the photosensitizer in the same nanoparticle system. Wu et al.^57^ designed a self-luminescent theranostic nanoreactor, which consisted of a bis(2,4,5-trichlorophenyl-6-carbopentoxyphenyl)oxalate (CPPO) chemical energy source, a PFPV CL converter, and a tetraphenylporphyrin (TPP) photosensitizer with NIR emission. The nanoreactor served as a specific H_2_O_2_ probe, allowing CPPO to react with H_2_O_2_ and eventually generate ^1^O_2_ and exhibit NIR CL through CRET. The abnormally high H_2_O_2_ in the tumor microenvironment (TME) triggered CL-involved photodynamic theranostics with NIR imaging and PDT treatment without limitations on penetration depth.

Compared with CL, BL is more common in organisms, including microorganisms, marine organisms, and some insects^58,59^. Yang et al.^59^ used the BL firefly luciferase system as the light source to activate photosensitizers for PDT. In this nanosystem, biodegradable poly(lactic-co-glycolic acid) nanoparticles were doped with the Rose Bengal (RB) photosensitizer and then conjugated with luciferase. *In vivo* experiments showed that the designed nanosystem effectively killed tumor cells and inhibited tumor growth through bioluminescence resonance energy transfer.

## Photothermal theranostics based on nanotechnology

Similar to PDT, PTT is another phototherapy strategy that requires additional irradiation. The principle of PTT is based on receiving light irradiation with specific wavelengths. The photothermal transducers transform light into heat, resulting in a hyperthermic microenvironment in the surrounding tumor tissues^60^. In addition to the therapeutic efficiency, the excellent optical properties of the photosensitizers or the nanocarriers can also be used for imaging^61^, therefore allowing imaging-guided photothermal theranostics.

### Nanoparticles functioning only as drug carriers

Like many other theranostic strategies, nanoparticles are usually used to co-deliver theranostic agents, enhance the stability of PTT agents, and prolong their blood circulation times^62,63^. Chen et al.^64^ designed a H_2_O_2_ responsible liposome nanoplatform for the co-delivery of horseradish peroxidase (HRP) and 2,2′-azino-bis(3-ethylbenzothiazoline-6-sulfonic acid) (ABTS). In the presence of H_2_O_2_, HRP was able to convert colorless ABTS into an oxidized form with NIR absorbance, which in turn enabled simultaneous PTT and PA imaging. With increased H_2_O_2_ concentration, elevated PA signals could be observed, enabling precise detection of H_2_O_2_ *in vivo*. Based on this characteristic, metastatic lymph nodes and non-metastatic lymph nodes with different H_2_O_2_ contents were distinguished by the nanoparticle Lipo@HRP&ABTS. In addition, because Lipo@HRP&ABTS was highly sensitive to H_2_O_2_, the PA imaging using Lipo@HRP&ABTS was used for the detection of brain gliomas, which were considered difficult because of the blood-brain barrier and the location of brain tumors.

### Nanoparticles functioning as both drug carriers and photothermal agents

Nanoparticles serving as both drug carriers and photothermal agents are usually inorganic materials, including semiconductors, upconversion nanoparticles, mesoporous carbon nanospheres, Au nanoparticles, carbon nanotubes, graphene, copper sulfide, and palladium^60,65–67^. BP has been recently shown to be an ideal candidate in photothermal theranostics. Unlike many other materials suffering from complicated preparation processes that limit mass production, BP can be easily obtained using exfoliation from bulk BP into thin BP sheets with a few layers or even a monolayer. In addition to high loading efficiency as a delivery platform, BP also possesses excellent photothermal conversion efficiency. Chen et al.^68^ obtained BP nanosheets with DOX delivery for PA imaging-guided combined PTT, PDT, and chemotherapy. The drug loading efficiency reached as high as 950%, which was higher than any other reported 2D drug carrier, which was attributed to the ultrahigh surface area of BP. In addition, the electronic interaction between DOX and BP also contributed to the excellent loading capacity. Further experiments indicated that the BP-DOX showed pH-dependent and irradiation-dependent drug releases, and the drug release could be promoted by the photothermal effect of BP. *In vivo* experiments on mice bearing 4T1 tumors also showed exceptional tumor growth inhibition when mice received BP-DOX with 660 nm and 808 nm laser irradiation.

**Figure 5:**
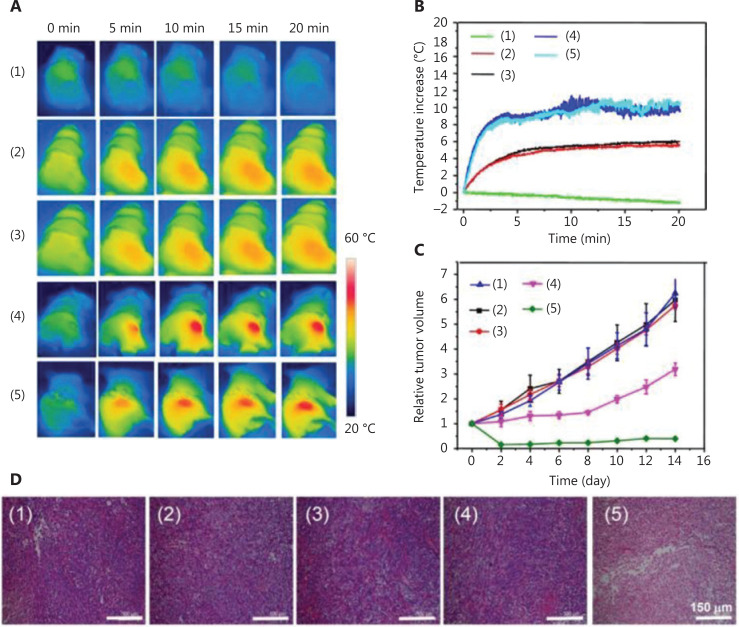
Schematic diagram of (A) IR thermal images of 4T1 tumor-bear mice treated with PDA-ICG-PEG/DOX (1), phosphate-buffered saline (plus a laser) (2), free doxorubicin (DOX) (plus a laser) (3), PDA-ICG-PEG (plus a laser) (4), and PDA-ICG-PEG/DOX (plus a laser) (5). (B) Temperature changes of tumor-bearing mice during laser irradiation. (C) Tumor growth curves. (D) Hematoxylin and eosin-stained slices of tumors collected from mice at 1 day post treatment. Reproduced with permission from Ref. 69.

In some cases, nanoparticles consisting of organic semiconducting polymers may also serve as photothermal transducers. Among these materials, polydopamine (PDA) nanoparticles are one of the most popular materials. Dong et al.^69^ designed a kind of PDA nanoparticle as a PTT agent, as well as a carrier for chemotherapeutic drugs. Considering the relatively low mass extinction coefficient in the NIR region for PDA as PTT agents, Indocyanine Green (ICG) was loaded into PDA nanoparticles, followed by modification of PEG. Compared with free ICG, the PDA-ICG-PEG nanoparticles exhibited red-shifted absorbance as well as increased photostability. To achieve a combination of chemotherapy and PTT, DOX was also loaded into PDA-ICG-PEG nanoparticles. The PDA-ICG-PEG/DOX nanoparticle could be imaged using MRI and imaging contrast agents, therefore allowing MRI imaging-guided combined therapy. *In vivo* studies of 4T1-tumor-bearing mice (**Figure 5**) showed that the PDA-ICG-PEG/DOX (plus laser) group performed best, with rapidly increased temperatures in tumor tissues, as well as for use in the smallest tumor volumes.

## Nanotheranostics-assisted gene therapy

It has been widely acknowledged that tumor occurrence, metastasis, angiogenesis, and proliferation are highly associated with relative gene expression^70^. Based on the complementary pairing nature of DNA and RNA, it is easy to achieve a specific targeting effect of oncological genes. RNA inference (RNAi) is an evolutionarily-conserved mechanism among eukaryotes to regulate the expression of target genes^71^. Double-stranded RNA (dsRNA)^72^ is unstable and can induce RNAi, suggesting a promising strategy for disease therapy through suppression of relevant genes. In recent years, RNAi-involved gene therapy has attracted increasing attention^73^. However, RNAi is still facing many limitations. Suitable nanotheranostics will greatly assist gene therapy in several ways, involving enhancing cellular uptake of RNA with negative charges^72^, and preventing RNA from biodegradation by RNase.

### Theranostics for siRNA therapy

SiRNAs are small interfering RNAs that can downregulate target genes by interfering with the expression of specific genes with complementary nucleotide sequences^74^. Kim et al.^74^ designed an aptamer-based targeting theranostic platform to co-delivery siRNA for gene therapy and QD for diagnosis. Anti-EGF receptor aptamer-lipid conjugates were inserted into the QD-lipid nanocarriers for triple negative breast cancer targeting (aptamo-QLs). The high absorption of aptamo-QLs by MDA-MB-231 cells, a cancer cell line with overexpressed EGF receptors, was confirmed by the fluorescence of labeled fluorescein isothiocynanate. *In vivo* experiments of mice using MDA-MB-231 xenografts showed that higher levels of red QD fluorescence in tumor tissues could be observed in aptamo-QLs, when compared with QL-treated groups. The aptamo-QLs co-delivering two therapeutic siRNAs was more effective in inhibiting tumor growth and recurrence.

### Theranostics for miRNA therapy

**Figure 6:**
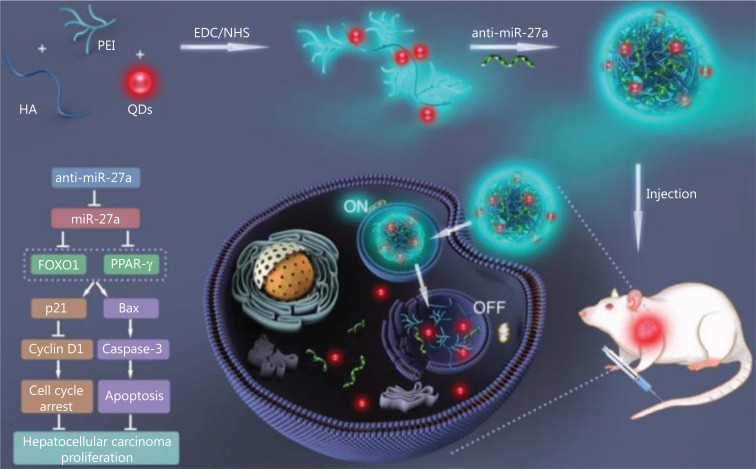
Schematic illustration of liver cancer theranostics based on the simultaneous bioimaging and miRNA-based modulation therapy enabled by anti-miR-27a/QD-HA-PEI. Reproduced with permission from Ref. 73.

MicroRNA (miRNA), a type of small endogenous noncoding RNA^75^, can reduce the expression of targeting genes^76^ at the post-transcriptional level^73^ via downregulating target mRNAs^77^. They are probably the most commonly used agents in silencing disease-associated genes. The miRNA is regarded as an ideal candidate to achieve theranostics of brain tumors^78^ and aggressive tumors like melanomas^79^. Yan et al.^80^ used polyethylenimine (PEI)-modified gold nanorods (AuNR-PEI) as the transport platform of fuel-improved miRNA explorer (FIRE), while the miRNA was used as the biomarker. Zheng et al.^73^ prepared the CD44-targeting delivery platform carrying anti-miR-27a for use in liver cancer theranostics (**Figure 6**). It has been reported that miR-27a was related to liver tumorigenesis and was overexpressed in both serum and liver tumor tissues^81^. Using encapsulated anti-miR-27a in QD-HA-PEI nanoparticles, a miRNA-based gene theranostic system was developed, based on the CD44-targeting property of HA and the NIR imaging property of QD. Both *in vitro* and *in vivo* experiments indicated the significant therapeutic effect of the designed nanosystem as well as excellent NIR fluorescence imaging.

### Theranostics for shRNA therapy

Similar to miRNA, short hairpin RNA (shRNA) involves one of the main nucleic acid therapeutics for cancer therapy^82^. Zhu et al.^83^ designed self-assembled intertwining DNA-RNA nanocapsules (iDR-NCs) that co-delivered CpG, Stat3 shRNA, and tumor-specific peptide neoantigens for enhanced immunotherapy. In addition to CpG, Janus kinase-signal transducer and activator of transcription (STAT) pathways were also shown to be related to cancer immunotherapy. Using incubation of iDR-NC-Alexa 555/CSIINFEK_(FITC)_L complexes, super resolution fluorescence imaging was used to detect its intracellular co-delivery, which showed that the co-delivery of iDR-NCs and CSIINFEK_(FITC)_L enhanced the cellular uptake of iDR-NCs and prolonged the presence of CSIINFEK_(FITC)_L on bone marrow derived dendritic cell surfaces. After administration of the iDR-NC/antigen, increased numbers of CD8^+^ T cells were detected with enhanced expression of programmed death receptor 1 (PD-1), showing an efficient modulation of the tumor immune microenvironment.

## “Cocktail” theranostics based on nanotechnology

Because of the insufficiency and different limitations of single oncotherapy, the combinational field of cancer therapies^84^,^85^ is gaining increased attention in basic and clinical studies. The administration of multimodal diagnostic and treatment strategies, namely “cocktail” theranostics, has become increasingly important in achieving the desired anticancer efficacies. We have discussed several combined theranostic strategies and platforms above, and in this section, we mainly focus on several “cocktail” theranostic systems involving recently developed chemotherapy and phototherapy.

### Chemotherapy-involved “cocktail” theranostics

Chemotherapy suffers from intrinsic or acquired drug resistance^84^, which significantly limits the therapeutic effects, so it needs to be used in combination with other therapies. For example, combining chemotherapeutic drugs with therapeutic nucleic acids can not only activate the immune system and regulate the expression of tumor-related genes through RNAi, but also overcome multidrug resistance^86^. In addition, co-delivering chemotherapeutic drugs and radionuclides also results in “cocktail” chemo-radiotheranostics. Zhong et al.^87^ fabricated PEG-modified PDA (PDA-PEG) for co-delivering both radionuclides and DOX to provide nuclear-imaging-guided combined radioisotope therapy and chemotherapy. After confirming the biocompatibility of PDA-PEG, the anti-tumor effects of ^131^I-PDA-PEG, PDA-PEG-DOX, and ^131^I-PDA-PEG-DOX were tested using *in vitro* experiments. ^131^I-PDA-PEG exhibited superior performance compared with free ^131^I, with effective destruction of tumor cells. In addition, *in vivo* experiments further validated the outstanding oncotherapeutic effects of combinational theranostics.

The hyperthermic TME during treatment can also enhance the diffusion of chemotherapeutic drugs^88^. Chen et al.^89^ used red blood cell (RBC) membranes to coat hollow Prussian blue nanoparticles (HMPB@RBC NPs) to deliver DOX. RBCs increased the biocompatibility and circulation of the nanoparticles, to escape clearance by the immune system as a foreign substance. In addition, the hollow mesoporous structure of the nanoparticles allowed a high DOX loading efficiency of up to 92%. Irradiating HMPB@RBC NPs with a 808 nm NIR laser for 5 min resulted in an effective temperature enhancement of approximately 30.2 °C. Both *in vitro* and *in vivo* experiments confirmed the excellent anti-tumor effect of HMPB@RBC NPs. Furthermore, HMPB NPs enabled ultrasound/PA dual-mode imaging, making HMPB@RBC NPs a promising strategy for “cocktail” theranostics.

### Phototherapy-involved “cocktail” theranostics

**Figure 7:**
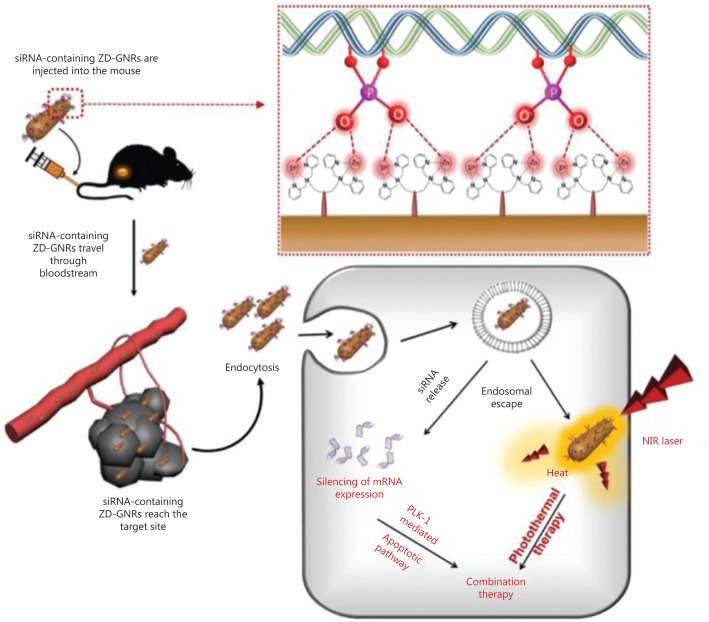
Schematic illustration of combined photothermal and gene therapy based on the specific interaction between the Zn(II)-dipicolylamine and phosphate groups of siRNA. Reproduced with permission from Ref. 90.

Min et al.^90^ used dipicolyl amine (DPA)-coated gold nanorods (GNR) containing Zn^2+^ (ZD-GNR) as a delivery system of anti-polo-like kinase 1 siRNA (siPLK) for PA imaging-guided combined PTT and gene therapy. PLK1 is one of serine/threonine protein kinases that plays an important role in the cell cycle. As a type of proto-oncogene, PLK1 is overexpressed in some types of cancers and serves as a typical target for gene therapy. The interaction between Zn^2+^ and the phosphates of RNA provides a high drug loading efficiency and also protects siRNA from degradation. Compared with the free Cy3-siRNA group, the Cy3-siRNA/LipoMax and siRNA/ZD-GNRs groups showed brighter red fluorescence in incubated 143B cancer cells, implying an enhanced cellular uptake due to the reduced negative charges of siRNA. *In vivo* experiments further showed the synergistic PTT-gene therapy efficacy with apparent inhibition of tumor growth (**Figure 7**).

Although both PDT and PTT have limitations such as the hypoxia microenvironment and thermal-resistance of residual cancer cells,^44^ the combination of PDT and PTT is expected to provide new opportunities for symbiotic effects in cancer treatment. Liu et al.^91^ reported that copper ferrite nanospheres (CFNs) had excellent performance in both enhanced ROS generation and PTT efficiency, as well as in MRI imaging. CFNs with 650 nm irradiation exhibited efficient ROS generation as well as excellent photothermal stability. Both *in vitro* and *in vivo* results validated successful tumor inhibition by combined PDT and PTT. In addition, due to the overlap of many PTT and radiotheranostic materials^92,93^, photothermal-radiotheranostics also holds great promise. Undoubtedly, there are more “cocktail” theranostic strategies than what we could summarize in this review, which also have exceptional performance in cancer treatments.

## Conclusions and prospects

In conclusion, the rapid development of nanotechnology-based theranostics has greatly promoted the revolution of cancer oncotherapy and diagnosis. Ideal nanotheranostic systems are expected to be (1) non-toxic and biocompatible, (2) highly stable and efficient for drug loading, (3) easy to prepare and modify; and (4) tumor targeting and effective for endocytosis^94,95^. Although most nanoparticles summarized above do not present obvious cytotoxicity, further experiments are necessary to ensure safety in clinical translations.^19^ In addition, the preparation and quality control of nanoparticles are still complicated. Especially, the synthesis of organic nanomaterials is typically laborious with low yields. Last, the balance between the efficiency and the reliability of nanotheranostic systems should be considered, although the introduction of multiple agents in one theranostic platform may bring diverse functions. However, the nanosystem could be too complicated for large-scale manufacturing. Nevertheless, current investigators have put increasing emphases on these issues, and a number of initiatives are being taken to achieve more practical applications of nanotheranostic platforms in cancer treatments.
